# (1R*,5S*)-8-(2-Fluoro-4-nitro­phen­yl)-8-aza­bicyclo­[3.2.1]octan-3-one

**DOI:** 10.1107/S1600536811047350

**Published:** 2011-11-12

**Authors:** Tao Yang, Jianzhong Yang, Zicheng Li, Youfu Luo

**Affiliations:** a State Key Laboratory of Biotherapy, West China Hospital, Sichuan University, Chengdu 610041, People’s Republic of China; b Department of Pharmaceutical and Bioengineering, School of Chemical Engineering, Sichuan University, Chengdu 610065, People’s Republic of China

## Abstract

In the title compound, C_13_H_13_FN_2_O_3_, the fused piperidine ring is in a chair conformation. The fused pyrrolidine ring shows an envelope conformation with the N atom displaced by 0.661 (3) Å out of the plane formed by the four C atoms of the pyrrolidine ring. The dihedral angle between this plane and the plane formed by the four attached C atoms of the piperidine ring (not including the carbonyl C atom) is 67.63 (10)°. The F atom is disordered and was refined using a split model with an occupancy ratio of 0.910 (3): 0.080 (3).

## Related literature

For a related structure, see Yang *et al.* (2008 ▶).
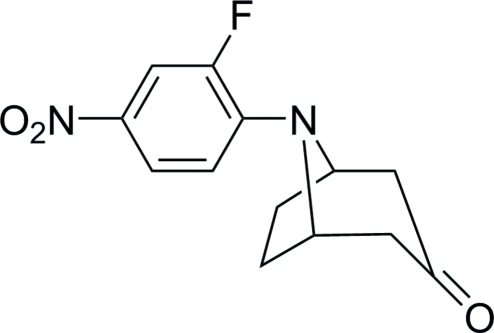


## Experimental

### 

#### Crystal data

C_13_H_13_FN_2_O_3_
*M* *_r_* = 264.25
Monoclinic, 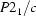

*a* = 7.2030 (3) Å
*b* = 11.3097 (4) Å
*c* = 14.8372 (6) Å
β = 97.391 (4)°
*V* = 1198.65 (8) Å^3^
*Z* = 4
Mo *K*α radiation
μ = 0.12 mm^−1^
*T* = 293 K
0.38 × 0.35 × 0.30 mm

#### Data collection

Agilent Xcalibur Eos diffractometer
Absorption correction: multi-scan (*CrysAlis PRO*; Agilent, 2010 ▶) *T*_min_ = 0.997, *T*_max_ = 1.0
4706 measured reflections
2109 independent reflections
1482 reflections with *I* > 2σ(*I*)
*R*_int_ = 0.016

#### Refinement

*R*[*F* ^2^ > 2σ(*F* ^2^)] = 0.044
*wR*(*F* ^2^) = 0.113
*S* = 1.02
2109 reflections
183 parameters
H-atom parameters constrained
Δρ_max_ = 0.14 e Å^−3^
Δρ_min_ = −0.12 e Å^−3^

### 

Data collection: *CrysAlis PRO* (Agilent, 2010 ▶); cell refinement: *CrysAlis PRO*; data reduction: *CrysAlis PRO*; program(s) used to solve structure: *SHELXS97* (Sheldrick, 2008 ▶); program(s) used to refine structure: *SHELXL97* (Sheldrick, 2008 ▶); molecular graphics: *OLEX2* (Dolomanov *et al.*, 2009 ▶); software used to prepare material for publication: *OLEX2*.

## Supplementary Material

Crystal structure: contains datablock(s) I, global. DOI: 10.1107/S1600536811047350/nc2245sup1.cif

Structure factors: contains datablock(s) I. DOI: 10.1107/S1600536811047350/nc2245Isup2.hkl

Supplementary material file. DOI: 10.1107/S1600536811047350/nc2245Isup3.cml

Additional supplementary materials:  crystallographic information; 3D view; checkCIF report

## Figures and Tables

**Fig. 1.**
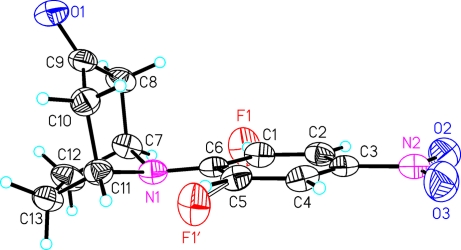
The molecular structure of the title compound, with labeling and displacement ellipsoids drawn at the 30% probability level. The disorder is shown as full and open bonds.

## supplementary crystallographic information

### Comment

The title compound is one important synthetic intermediates in our efforts to synthesize oxazolidinone derivatives. To identify< this compound its single crystal structure was determined by single crystal X-ray diffraction.

The dihedral angle between the benzene ring and the plane built up of C7, C8, C10 and C11 of the piperidine ring is 86.59 (9)° while the angle between the plane defined by this four C atoms and the plane formed by the four C atoms of the pyrrolidine ring is 67.63 (10)°. The fused piperidine ring is in a chair conformation with the N atom and one C atom displaced by 0.8433 (26) Å and -0.3798 (33) Å out of the mean plane defined by the other four atoms. The fused pyrrolidine ring adopts an envelope conformation with the N atom deviating by 0.661 (3) Å.

### Experimental

A solution of 1,2-difluoro-4-nitrobenzene (2.5 g, 15.7 mmol), nortropinone hydrochloride (3.8 g, 23.6 mmol) and anhydrous potassium carbonate (4.3 g,31.4 mmol) in DMF (75 mL) was stirred at 100°C for 2 h. Water (300 ml) was added and precipitation was formed. (1*R*, 5S)-8-(2-fluoro-4-nitrophenyl)-8- azabicyclo[3.2.1]octan-3-one was collected by filtration and recrystallized from ethylacetate.Crystals suitable for X-ray analysis were obtained by slow evaporation from a solution of the title compound in acetone.

### Refinement

All H atoms were positioned with idealized geometry (C—H = 0.93-0.98 Å). and were refined using a riding model, with *U*ĩso(H) = 1.2*U*eq(C). The fluoro atom is disordered in two orientations and was refined using a split model and sof of 0.910:0.099 (3).

### Crystal data

**Table:**

C_13_H_13_FN_2_O_3_	*F*(000) = 552
*M**_r_* = 264.25	*D*_x_ = 1.464 Mg m^−^^3^
Monoclinic, *P*2_1_/*c*	Mo *K*α radiation, λ = 0.7107 Å
Hall symbol: -P 2ybc	Cell parameters from 1673 reflections
*a* = 7.2030 (3) Å	θ = 3.0–28.9°
*b* = 11.3097 (4) Å	µ = 0.12 mm^−^^1^
*c* = 14.8372 (6) Å	*T* = 293 K
β = 97.391 (4)°	Block, yellow
*V* = 1198.65 (8) Å^3^	0.38 × 0.35 × 0.30 mm
*Z* = 4	

### Data collection

**Table:**

Agilent Xcalibur Eos diffractometer	2109 independent reflections
Radiation source: fine-focus sealed tube	1482 reflections with *I* > 2σ(*I*)
graphite	*R*_int_ = 0.016
Detector resolution: 16.0874 pixels mm^-1^	θ_max_ = 25.0°, θ_min_ = 3.3°
ω scans	*h* = −8→8
Absorption correction: multi-scan (*CrysAlis PRO*; Agilent, 2010)	*k* = −8→13
*T*_min_ = 0.997, *T*_max_ = 1.0	*l* = −17→14
4706 measured reflections	

### Refinement

**Table:**

Refinement on *F*^2^	Secondary atom site location: difference Fourier map
Least-squares matrix: full	Hydrogen site location: inferred from neighbouring sites
*R*[*F*^2^ > 2σ(*F*^2^)] = 0.044	H-atom parameters constrained
*wR*(*F*^2^) = 0.113	*w* = 1/[σ^2^(*F*_o_^2^) + (0.0452*P*)^2^ + 0.1579*P*] where *P* = (*F*_o_^2^ + 2*F*_c_^2^)/3
*S* = 1.02	(Δ/σ)_max_ < 0.001
2109 reflections	Δρ_max_ = 0.14 e Å^−^^3^
183 parameters	Δρ_min_ = −0.12 e Å^−^^3^
0 restraints	Extinction correction: *SHELXL97* (Sheldrick, 2008), Fc^*^=kFc[1+0.001xFc^2^λ^3^/sin(2θ)]^-1/4^
Primary atom site location: structure-invariant direct methods	Extinction coefficient: 0.0037 (11)

### Special details

**Table:**

Geometry. All e.s.d.'s (except the e.s.d. in the dihedral angle between two l.s. planes) are estimated using the full covariance matrix. The cell e.s.d.'s are taken into account individually in the estimation of e.s.d.'s in distances, angles and torsion angles; correlations between e.s.d.'s in cell parameters are only used when they are defined by crystal symmetry. An approximate (isotropic) treatment of cell e.s.d.'s is used for estimating e.s.d.'s involving l.s. planes.
Refinement. Refinement of *F*^2^ against ALL reflections. The weighted *R*-factor *wR* and goodness of fit *S* are based on *F*^2^, conventional *R*-factors *R* are based on *F*, with *F* set to zero for negative *F*^2^. The threshold expression of *F*^2^ > σ(*F*^2^) is used only for calculating *R*-factors(gt) *etc*. and is not relevant to the choice of reflections for refinement. *R*-factors based on *F*^2^ are statistically about twice as large as those based on *F*, and *R*- factors based on ALL data will be even larger.

### Fractional atomic coordinates and isotropic or equivalent isotropic displacement parameters (Å^2^)

**Table:**

	*x*	*y*	*z*	*U*_iso_*/*U*_eq_	Occ. (<1)
F1	0.6635 (2)	0.70834 (11)	0.61012 (10)	0.0924 (6)	0.910 (3)
F1'	0.716 (2)	0.2944 (9)	0.6621 (9)	0.080 (6)	0.090 (3)
O1	1.1163 (2)	0.57062 (17)	0.92502 (11)	0.0972 (6)	
O2	0.7844 (3)	0.54056 (17)	0.32301 (12)	0.0929 (6)	
O3	0.8052 (3)	0.35184 (18)	0.34414 (12)	0.1019 (6)	
N1	0.6650 (2)	0.51844 (13)	0.73584 (11)	0.0530 (4)	
N2	0.7860 (2)	0.4527 (2)	0.37182 (13)	0.0706 (6)	
C1	0.7027 (3)	0.59691 (16)	0.58549 (15)	0.0568 (5)	
H1	0.6815	0.6733	0.6051	0.068*	0.090 (3)
C2	0.7289 (3)	0.58145 (18)	0.49751 (15)	0.0592 (6)	
H2	0.7253	0.6459	0.4584	0.071*	
C3	0.7608 (3)	0.46959 (18)	0.46658 (14)	0.0534 (5)	
C4	0.7681 (3)	0.37456 (18)	0.52508 (14)	0.0583 (6)	
H4	0.7901	0.2988	0.5044	0.070*	
C5	0.7427 (3)	0.39274 (16)	0.61379 (14)	0.0537 (5)	
H5	0.7504	0.3280	0.6528	0.064*	0.910 (3)
C6	0.7055 (2)	0.50442 (15)	0.64893 (13)	0.0469 (5)	
C7	0.6776 (3)	0.63004 (17)	0.78822 (14)	0.0608 (6)	
H7	0.6162	0.6951	0.7523	0.073*	
C8	0.8836 (3)	0.65642 (18)	0.81689 (15)	0.0659 (6)	
H8A	0.9432	0.6734	0.7633	0.079*	
H8B	0.8948	0.7262	0.8552	0.079*	
C9	0.9831 (3)	0.5552 (2)	0.86761 (14)	0.0648 (6)	
C10	0.9161 (3)	0.43248 (18)	0.84129 (14)	0.0627 (6)	
H10B	0.9423	0.3808	0.8936	0.075*	
H10A	0.9857	0.4031	0.7942	0.075*	
C11	0.7064 (3)	0.42723 (17)	0.80686 (13)	0.0568 (5)	
H11	0.6703	0.3485	0.7832	0.068*	
C12	0.5736 (3)	0.5997 (2)	0.86856 (17)	0.0799 (7)	
H12B	0.6325	0.6376	0.9236	0.096*	
H12A	0.4443	0.6254	0.8571	0.096*	
C13	0.5847 (3)	0.4658 (2)	0.87766 (16)	0.0720 (7)	
H13B	0.6410	0.4432	0.9381	0.086*	
H13A	0.4612	0.4306	0.8658	0.086*	

### Atomic displacement parameters (Å^2^)

**Table:**

	*U*^11^	*U*^22^	*U*^33^	*U*^12^	*U*^13^	*U*^23^
F1	0.1396 (15)	0.0391 (8)	0.1023 (11)	0.0162 (8)	0.0297 (10)	0.0065 (7)
F1'	0.137 (14)	0.028 (7)	0.074 (9)	−0.001 (7)	0.017 (8)	0.013 (6)
O1	0.0642 (11)	0.1378 (16)	0.0858 (12)	−0.0305 (10)	−0.0049 (9)	−0.0118 (11)
O2	0.0934 (14)	0.1075 (14)	0.0781 (11)	−0.0261 (11)	0.0124 (9)	0.0127 (10)
O3	0.1195 (17)	0.0967 (14)	0.0929 (13)	−0.0086 (12)	0.0269 (11)	−0.0254 (11)
N1	0.0507 (10)	0.0397 (9)	0.0676 (11)	−0.0017 (7)	0.0040 (8)	−0.0014 (8)
N2	0.0501 (11)	0.0876 (16)	0.0732 (14)	−0.0159 (11)	0.0048 (9)	−0.0051 (12)
C1	0.0535 (13)	0.0351 (11)	0.0812 (15)	0.0038 (9)	0.0066 (11)	0.0018 (10)
C2	0.0477 (12)	0.0523 (13)	0.0765 (15)	−0.0031 (10)	0.0037 (10)	0.0138 (11)
C3	0.0349 (11)	0.0593 (13)	0.0644 (13)	−0.0056 (9)	0.0004 (9)	−0.0025 (10)
C4	0.0481 (12)	0.0488 (12)	0.0749 (15)	0.0017 (9)	−0.0036 (10)	−0.0074 (11)
C5	0.0478 (12)	0.0424 (11)	0.0676 (13)	0.0024 (9)	−0.0050 (10)	0.0017 (10)
C6	0.0330 (10)	0.0399 (11)	0.0657 (13)	0.0002 (8)	−0.0019 (9)	−0.0013 (9)
C7	0.0551 (13)	0.0461 (12)	0.0833 (15)	0.0002 (10)	0.0166 (11)	−0.0080 (10)
C8	0.0647 (15)	0.0551 (13)	0.0810 (15)	−0.0168 (11)	0.0210 (11)	−0.0181 (11)
C9	0.0432 (12)	0.0883 (17)	0.0649 (14)	−0.0143 (12)	0.0142 (10)	−0.0139 (12)
C10	0.0553 (13)	0.0660 (14)	0.0661 (13)	0.0060 (11)	0.0048 (10)	0.0038 (10)
C11	0.0557 (13)	0.0466 (11)	0.0664 (13)	−0.0072 (10)	0.0019 (10)	0.0023 (10)
C12	0.0624 (15)	0.0776 (17)	0.1054 (19)	−0.0054 (13)	0.0326 (13)	−0.0145 (14)
C13	0.0540 (14)	0.0802 (17)	0.0827 (16)	−0.0116 (12)	0.0121 (11)	0.0040 (13)

### Geometric parameters (Å, °)

**Table:**

F1—C1	1.352 (2)	C5—C6	1.405 (3)
F1'—C5	1.350 (11)	C7—H7	0.9800
O1—C9	1.211 (2)	C7—C8	1.519 (3)
O2—N2	1.229 (2)	C7—C12	1.526 (3)
O3—N2	1.226 (2)	C8—H8A	0.9700
N1—C6	1.368 (2)	C8—H8B	0.9700
N1—C7	1.479 (2)	C8—C9	1.501 (3)
N1—C11	1.477 (2)	C9—C10	1.504 (3)
N2—C3	1.453 (3)	C10—H10B	0.9700
C1—H1	0.9300	C10—H10A	0.9700
C1—C2	1.354 (3)	C10—C11	1.532 (3)
C1—C6	1.405 (3)	C11—H11	0.9800
C2—H2	0.9300	C11—C13	1.516 (3)
C2—C3	1.375 (3)	C12—H12B	0.9700
C3—C4	1.378 (3)	C12—H12A	0.9700
C4—H4	0.9300	C12—C13	1.522 (3)
C4—C5	1.368 (3)	C13—H13B	0.9700
C5—H5	0.9300	C13—H13A	0.9700
			
F1—C1—C2	116.15 (18)	C6—C1—H1	118.1
F1—C1—C6	119.9 (2)	C6—C5—H5	118.4
F1'—C5—C4	115.6 (6)	C7—C8—H8A	109.2
F1'—C5—H5	12.4	C7—C8—H8B	109.2
F1'—C5—C6	119.7 (6)	C7—C12—H12B	110.6
O1—C9—C8	121.8 (2)	C7—C12—H12A	110.6
O1—C9—C10	120.9 (2)	C8—C7—H7	111.2
O2—N2—C3	118.1 (2)	C8—C7—C12	112.66 (19)
O3—N2—O2	123.2 (2)	C8—C9—C10	117.21 (18)
O3—N2—C3	118.7 (2)	H8A—C8—H8B	107.9
N1—C6—C1	123.97 (17)	C9—C8—C7	112.12 (18)
N1—C6—C5	121.87 (17)	C9—C8—H8A	109.2
N1—C7—H7	111.2	C9—C8—H8B	109.2
N1—C7—C8	107.84 (16)	C9—C10—H10B	109.0
N1—C7—C12	102.44 (17)	C9—C10—H10A	109.0
N1—C11—C10	108.09 (16)	C9—C10—C11	113.06 (17)
N1—C11—H11	111.1	C10—C11—H11	111.1
N1—C11—C13	102.23 (16)	H10B—C10—H10A	107.8
C1—C2—H2	120.3	C11—N1—C7	103.18 (15)
C1—C2—C3	119.37 (19)	C11—C10—H10B	109.0
C2—C1—H1	118.1	C11—C10—H10A	109.0
C2—C1—C6	123.89 (18)	C11—C13—C12	104.67 (18)
C2—C3—N2	119.4 (2)	C11—C13—H13B	110.8
C2—C3—C4	120.1 (2)	C11—C13—H13A	110.8
C3—C2—H2	120.3	C12—C7—H7	111.2
C3—C4—H4	120.3	C12—C13—H13B	110.8
C4—C3—N2	120.5 (2)	C12—C13—H13A	110.8
C4—C5—H5	118.4	H12B—C12—H12A	108.8
C4—C5—C6	123.22 (19)	C13—C11—C10	113.00 (17)
C5—C4—C3	119.33 (19)	C13—C11—H11	111.1
C5—C4—H4	120.3	C13—C12—C7	105.51 (18)
C5—C6—C1	114.05 (19)	C13—C12—H12B	110.6
C6—N1—C7	126.04 (16)	C13—C12—H12A	110.6
C6—N1—C11	122.93 (16)	H13B—C13—H13A	108.9
			
F1—C1—C2—C3	−177.08 (17)	C6—N1—C7—C8	−74.0 (2)
F1—C1—C6—N1	2.1 (3)	C6—N1—C7—C12	167.00 (18)
F1—C1—C6—C5	178.26 (17)	C6—N1—C11—C10	77.6 (2)
F1'—C5—C6—N1	8.4 (8)	C6—N1—C11—C13	−162.92 (17)
F1'—C5—C6—C1	−167.9 (8)	C6—C1—C2—C3	−0.1 (3)
O1—C9—C10—C11	152.0 (2)	C7—N1—C6—C1	−23.0 (3)
O2—N2—C3—C2	2.6 (3)	C7—N1—C6—C5	161.09 (17)
O2—N2—C3—C4	−177.66 (18)	C7—N1—C11—C10	−73.0 (2)
O3—N2—C3—C2	−176.46 (19)	C7—N1—C11—C13	46.42 (19)
O3—N2—C3—C4	3.3 (3)	C7—C8—C9—O1	−150.3 (2)
N1—C7—C8—C9	−54.7 (2)	C7—C8—C9—C10	32.5 (3)
N1—C7—C12—C13	23.7 (2)	C7—C12—C13—C11	4.1 (2)
N1—C11—C13—C12	−30.4 (2)	C8—C7—C12—C13	−91.9 (2)
N2—C3—C4—C5	−179.57 (16)	C8—C9—C10—C11	−30.7 (3)
C1—C2—C3—N2	179.04 (17)	C9—C10—C11—N1	50.9 (2)
C1—C2—C3—C4	−0.7 (3)	C9—C10—C11—C13	−61.5 (2)
C2—C1—C6—N1	−174.79 (18)	C10—C11—C13—C12	85.5 (2)
C2—C1—C6—C5	1.4 (3)	C11—N1—C6—C1	−166.83 (16)
C2—C3—C4—C5	0.2 (3)	C11—N1—C6—C5	17.3 (3)
C3—C4—C5—F1'	167.7 (8)	C11—N1—C7—C8	75.5 (2)
C3—C4—C5—C6	1.2 (3)	C11—N1—C7—C12	−43.6 (2)
C4—C5—C6—N1	174.33 (17)	C12—C7—C8—C9	57.6 (2)
C4—C5—C6—C1	−2.0 (3)		
